# Impact of diagnosis to ablation time on clinical outcomes in patients with atrial fibrillation: post hoc analysis of the CABANA trial

**DOI:** 10.1186/s12916-026-04615-3

**Published:** 2026-01-12

**Authors:** Manlin Zhao, Yang Chen, Mingxiao Li, Chao Jiang, Zhen Wang, Hongyu Liu, Liu He, Caihua Sang, Xin Du, Jianzeng Dong, Douglas L. Packer, Changsheng Ma, Gregory Y. H. Lip

**Affiliations:** 1 Department of Cardiology, Beijing Anzhen Hospital, Capital Medical University, National Clinical Research Centre for Cardiovascular Diseases, Beijing, China; 2 Liverpool Centre for Cardiovascular Science at University of Liverpool, Liverpool John Moores University and Liverpool Heart and Chest Hospital, Liverpool, UK; 3 Department of Cardiovascular and Metabolic Medicine, Institute of Life Course and Medical Sciences, University of Liverpool, Liverpool, UK; 4 Department of Cardiovascular Medicine, the Second Affiliated Hospital, Jiangxi Medical College, Nanchang University, Jiangxi, China; 5 Beijing Advanced Innovation Centre for Big Data-Based Precision Medicine for Cardiovascular Diseases, Beijing, China; 6 Heart Health Research Centre, Beijing, China; 7 The First Affiliated Hospital of Zhengzhou University, Zhengzhou, China; 8 Mayo Clinic, Rochester, MN USA; 9 Engineering Research Centre of Medical Devices for Cardiovascular Diseases, Ministry of Education, Beijing, China; 10 Danish Centre for Health Services Research, Department of Clinical Medicine, Aalborg University, Aalborg, Denmark; 11 Department of Cardiology, Lipidology and Internal Medicine, Medical University of Bialystok, Bialystok, Poland

**Keywords:** Atrial fibrillation, Catheter ablation, Diagnosis-to-ablation time, Recurrence, Prognosis

## Abstract

**Background:**

Current evidence is limited concerning the temporal impact of atrial fibrillation (AF) ablation timings on post-ablation outcomes.

**Methods:**

Patients who experienced ablation in the CABANA trial were enrolled in our analysis. Diagnosis to ablation time (DAT) was calculated from the date when the initial AF episode was documented. The primary endpoint was a composite of death, disabling stroke, serious bleeding or cardiac arrest. Secondary end points included AF recurrence, all-cause mortality, and all-cause mortality or cardiovascular hospitalisation. Associations between DAT and post-ablation outcomes were evaluated by restricted cubic spline (RCS) curves based on Cox models.

**Results:**

Out of 1145 patients (median age 67.0 years, interquartile range (IQR) 61.0–72.0; 36.7% female) included, 538 (47.0%) underwent early ablation (DAT ≤ 1 year), including 242 (45.0%) who received very early ablation (DAT ≤ 90 days). The overall median DAT was 423 days (IQR 118–1473). The L-shaped association was observed between DAT and the primary outcome (*P* for non-linear = 0.034). The lowest point was located at a DAT of approximately 1 to 3 years. For AF recurrences, the RCS curve rose progressively with increasing DAT (*P* for non-linear = 0.062), showing a reduced risk when DAT was less than 1 year. Effects of early ablation on the primary outcome favoured patients with initial AAD use (HR 0.86, 95% confidence interval (CI) 0.43–1.73 vs. HR 2.20, 95% CI *p*-interaction = 0.045).

**Conclusions:**

The benefits of earlier ablation in reducing AF recurrence might not equate to improvements in post-ablation cardiovascular prognosis. Timely treatment combining AADs and ablation may provide additional cardiovascular benefits.

**Trial registration:**

ClinicalTrials.gov Identifier: NCT00911508.

**Supplementary Information:**

The online version contains supplementary material available at 10.1186/s12916-026-04615-3.

## Background

Rhythm control is one option for consideration as part of the holistic management of patients with atrial fibrillation (AF) [1]. Rhythm control has been shown to improve cardiovascular (CV) outcomes in selected patients [2, 3], with AF catheter ablation (AFCA) demonstrating superiority over antiarrhythmic drug (AAD) in reducing recurrence and CV hospitalisations [4–6]. Nevertheless, its long-term efficacy still remains suboptimal, with recurrence rates reaching up to 50% [7].

AF is a progressive condition driven by structural and electrophysiological remodelling [8–10], which is believed to play a key role in promoting AF recurrence [11]. One potential marker of disease progression is the diagnosis to ablation time (DAT), as it may reflect the extent of atrial remodelling. Although direct evidence linking DAT to the degree of atrial remodelling remains limited, emerging evidence has indicated that a shorter DAT is associated with a reduced risk of post-ablation AF recurrence. As a result, DAT has been increasingly recognised as a critical predictor of ablation efficacy [12–14].

Nevertheless, evidence regarding the effect of DAT on CV outcomes is still insufficient. Increased time in sinus rhythm has been shown to be associated with improved mortality and CV outcomes [15]. Therefore, reducing DAT, which may increase time in sinus rhythm by reducing the risk of AF recurrence, is expected to be linked to better prognosis. Some observational studies have reported reduced risks of ischaemic stroke, heart failure (HF), hospitalisation, and all-cause mortality when DAT ≤ 1 year [12, 16, 17]. Whereas, similar CV benefits were not observed when DAT ≤ 3 years in another study. It remains unclear whether the prognostic benefits of early ablation diminish progressively as DAT increases, or if an optimal threshold exists for better post-ablation outcomes.

To address this gap, we analysed data from the CABANA (Catheter Ablation vs. Antiarrhythmic Drug Therapy for Atrial fibrillation) randomised controlled trial (RCT), which enrolled a diverse AF population with varying AF types and comorbidity burdens. Our aim was to assess the impact of DAT on trial-adjudicated AF recurrence and post-ablation CV outcomes and to identify potential temporal changes in these effects.

## Methods

The design, process, and results of the CABANA trial have been previously published [18–20]. Briefly, the CABANA trial was a multicentre randomised controlled trial conducted across 126 centres in 10 countries. From November 2009 to April 2016, the trial enrolled 2204 participants with new-onset or undertreated AF who were randomly assigned to receive AFCA versus drug therapy. Enrolled patients were aged ≥ 65 years, or younger than 65 years with one or more stroke risk factors. Patients were excluded if they had a prior left atrial AFCA or had failed ≥ 2 AAD. Ablation procedures were performed by physicians with ≥ 100 prior cases, and all patients received pulmonary vein isolation. Ancillary ablation techniques were used at the investigator’s discretion. All patients provided written informed consent, and participating sites received approval from their institutional review board or ethics committee to participate.

### Study population and design

For this analysis, we included patients receiving AFCA, comprising both those initially randomised to the ablation arm and those who crossed over from the drug therapy arm to ablation. Patients who crossed over from the ablation to the drug therapy arm were excluded. The date of the first AF onset was defined as the date of the initial documented episode. For patients initially assigned to the ablation arm, the DAT was considered the duration of AF prior to enrolment. For those initially assigned to the drug therapy arm but later crossing over to the ablation, the DAT was calculated by adding the duration of AF prior to enrolment to the time from enrolment to ablation.

Patients were grouped into four categories based on their DAT: very early AFCA (0 ≤ DAT ≤ 90 days), early AFCA (90 days < DAT ≤ 1 year), intermediate AFCA (1 year < DAT ≤ 3 year), and late AFCA (DAT > 3 years). This grouping was informed by prevailing research standards, which typically use 1 or 3 years as cut-off points for classifying ablation timing. Due to the absence of a definition for very early AFCA, we considered patients with DAT ≤ 90 days as having received very early AFCA, based on its clinical significance suggested by previous clinical evidence [21].

### Outcomes

The CABANA primary outcome was a composite of total mortality, disabling stroke, serious bleeding, or cardiac arrest. Three non-CABANA secondary end points to be analysed in our research included AF recurrence, all-cause mortality, all-cause mortality or CV hospitalisation. The CABANA Clinical Events Committee provided a blinded adjudication of the events comprising the primary endpoint [19].

The AF recurrence was defined as an episode of AF lasting ≥ 30 s beyond the 90-day blanking period, detected using a proprietary electrocardiogram (ECG) monitoring system (Medicomp, Inc., Florida). AF recurrences were adjudicated by the CABANA ECG Core laboratory [22]. In our study, a total of 733 patients were evaluated for AF recurrence using a proprietary ECG monitoring system.

### Statistical analysis

Continuous variables were presented as median (interquartile range, IQR); categorical variables were reported as percentages. Baseline differences among the groups were examined by Kruskal–Wallis test for continuous variables and chi-square test for categorical variables.

Associations between continuous DAT and clinical outcomes were evaluated by restricted cubic spline (RCS) curves with four knots based on Cox proportional hazards regression models. Covariates include age (≥ 65 years), sex, CHA_2_DS_2_-VA scores [23], paroxysmal AF (PAF) and use of oral anticoagulants (OAC) at baseline. Multiple-adjusted Cox models were further used to assess the associations between DAT categories and clinical outcomes, adjusting for the same covariates. The proportional hazards assumption was tested using the Schoenfeld residual test. Cumulative event rates for the primary outcome were calculated with Kaplan–Meier curves.

We conducted two sensitivity analyses: (1) to account for potential heterogeneity between patients randomised to the ablation arm and those who crossed over from the drug therapy arm, we repeated the multivariable Cox analyses exclusively among 998 patients randomised to the ablation arm; (2) to adjust for potential confounding effects related to differences in baseline HF prevalence among the four groups, we further included baseline HF history as a covariate in the previously established multiple-adjusted Cox models.

For subgroup analyses, DAT was dichotomised into early (≤ 1 year) and delayed groups (> 1 year). Cox models were performed among different subgroups based on age (≥ 65 years), sex, HF, AAD use, AF severity (Canadian Cardiovascular Society symptom of atrial fibrillation level 0–I vs. II–IV), and OAC use. Interaction terms were included to assess whether the effect of early ablation differed by subgroup.

The threshold for statistical significance was *p* < 0.05. The hypothesis test was two-sided. All statistical analyses were performed with the R software (R Core Team [2024]. R: A Language and Environment for Statistical Computing. R Foundation for Statistical Computing, Vienna, Austria; https://www.R-project.org/).

## Results

### Baseline characteristics

**Fig. 1:**
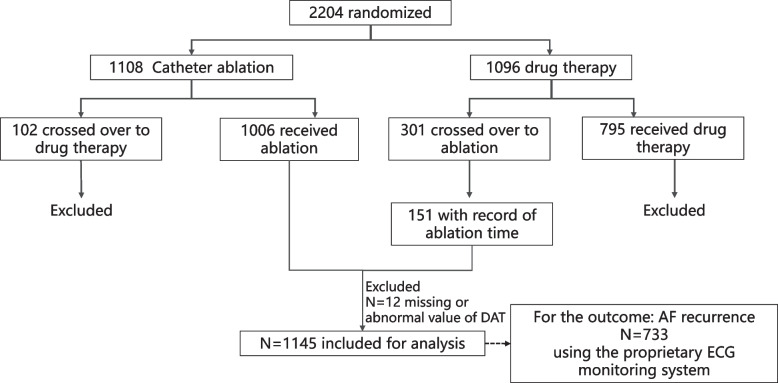
Study flowchart

A total of 1145 patients were included in the study (Fig. 1), with a median age of 67.0 years (IQR 61.0–72.0), and 36.7% were female. Among them, 538 (47.0%) underwent ablation within 1 year of AF diagnosis, including 242 (45.0%) who received very early AFCA (DAT ≤ 90 days).

The overall median DAT was 423 days (IQR 118–1473). Baseline characteristics stratified by DAT are presented in Table 1. No significant differences were observed across groups in terms of age, sex, body mass index, CHA_2_DS_2_-VASc/CHA_2_DS_2_-VA scores, OAC use or comorbidities including hypertension, diabetes, cerebral vascular accident or transient ischaemic attack, and coronary artery disease. Patients in the very early AFCA group had a higher prevalence of HF. A greater proportion of patients with persistent AF received ablation within 1 year of diagnosis, whereas this was less common among those with long-standing persistent AF. Over half of patients (55.4%) in the late AFCA group had prior or current AAD use, the highest proportion among all groups. Proportions of patients with previous or current specific AAD use at baseline were shown in Additional file 1: Table S1.

**Table 1:** Baseline characteristics

	Very early AFCA (0 ≤ DAT ≤ 90 days) (*N* = 242)	Early AFCA (90 days < DAT ≤ 1 year) (*N* = 296)	Intermediate AFCA (1 year < DAT ≤ 3 years) (*N* = 248)	Late AFCA (DAT > 3 years) (*N* = 359)	*P* value
Age, years	68.0 [63.0, 72.8]	68.0 [61.0, 71.0]	68.0 [60.0, 71.0]	66.0 [61.0, 71.0]	0.106
Male gender (%)	148 (61.2)	198 (66.9)	156 (62.9)	223 (62.1)	0.502
BMI, kg/m^2^	29.8 [26.4, 34.8]	30.3 [26.7, 34.1]	31.1 [27.1, 34.3]	29.6 [26.2, 33.9]	0.161
AF severity (CCS class)					0.208
Class 0	34 (14.1)	27 (9.2)	20 (8.1)	22 (6.1)	
Class I	37 (15.4)	43 (14.6)	34 (13.8)	49 (13.7)	
Class II	63 (26.1)	90 (30.5)	81 (32.9)	121 (33.8)	
Class III	92 (38.2)	110 (37.3)	95 (38.6)	135 (37.7)	
Class IV	15 (6.2)	25 (8.5)	16 (6.5)	31 (8.7)	
Hypertension	194 (80.2)	229 (77.4)	203 (81.9)	276 (76.9)	0.422
Diabetes	59 (24.4)	72 (24.3)	62 (25.0)	98 (27.3)	0.797
Prior CVA or TIA	27 (11.2)	25 (8.4)	32 (12.9)	41 (11.4)	0.396
Coronary artery disease	47 (19.4)	61 (20.6)	46 (18.5)	70 (19.5)	0.946
Perivascular diseases	11 (4.5)	7 (2.4)	13 (5.2)	18 (5.0)	0.293
Sleep apnoea	49 (20.2)	74 (25.0)	61 (24.6)	92 (25.6)	0.460
Family history of AF	20 (8.3)	41 (13.9)	23 (9.3)	49 (13.7)	0.075
Heart failure	50 (20.7)	42 (14.2)	36 (14.5)	39 (10.9)	0.011
LVEF	58.5 [51.8, 64.0]	57.5 [53.8, 62.0]	59.0 [55.0, 62.0]	60.0 [55.0, 65.0]	0.285
LVEF ≤ 35%	13 (8.3)	10 (5.4)	5 (3.0)	9 (3.7)	0.106
AF type at enrolment					< 0.001
Paroxysmal	103 (42.6)	127 (42.9)	99 (39.9)	173 (48.2)	
Persistent	133 (55.0)	156 (52.7)	106 (42.7)	138 (38.4)	
Long-standing persistent	6 (2.5)	13 (4.4)	43 (17.3)	48 (13.4)	
CHA_2_DS_2_-VASc score	3.0 [2.0, 4.0]	3.0 [2.0, 4.0]	3.0 [2.0, 3.0]	3.0 [2.0, 3.0]	0.070
CHA_2_DS_2_-VASc score ≥ 2	204 (84.3)	239 (80.7)	200 (80.6)	293 (81.6)	0.691
CHA_2_DS_2_-VA score	2.0 [2.0, 3.0]	2.0 [2.0, 3.0]	2.0 [1.0, 3.0]	2.0 [2.0, 3.0]	0.096
CHA_2_DS_2_-VA score ≥ 2	195 (80.6)	223 (75.3)	185 (74.6)	272 (75.8)	0.383
Current/previous OAC use	169 (69.8)	222 (75.0)	197 (79.4)	275 (76.6)	0.089
Current/previous AAD use	83 (34.3)	125 (42.2)	102 (41.1)	199 (55.4)	< 0.01

Categorical variables are presented as *n* (%). Continuous variables are presented median (Q1–Q3)

*Abbreviations*: *DAT* diagnosis to ablation time, *BMI* body mass index, *AF* atrial fibrillation, *CCS* Canadian Cardiovascular Society, *CVA* cerebral vascular accident, *TIA* transient ischaemic attack, *LVEF* left ventricular ejection fraction, *AAD* antiarrhythmic drug

### Associations between continuous DAT change and clinical outcomes

During a median follow-up of 1509 (IQR 952–1870) days, there were 84 primary outcome events among the overall population. Among 733 patients evaluated by the proprietary ECG monitoring system, 419 cases of AF recurrence were determined.

**Fig. 2:**
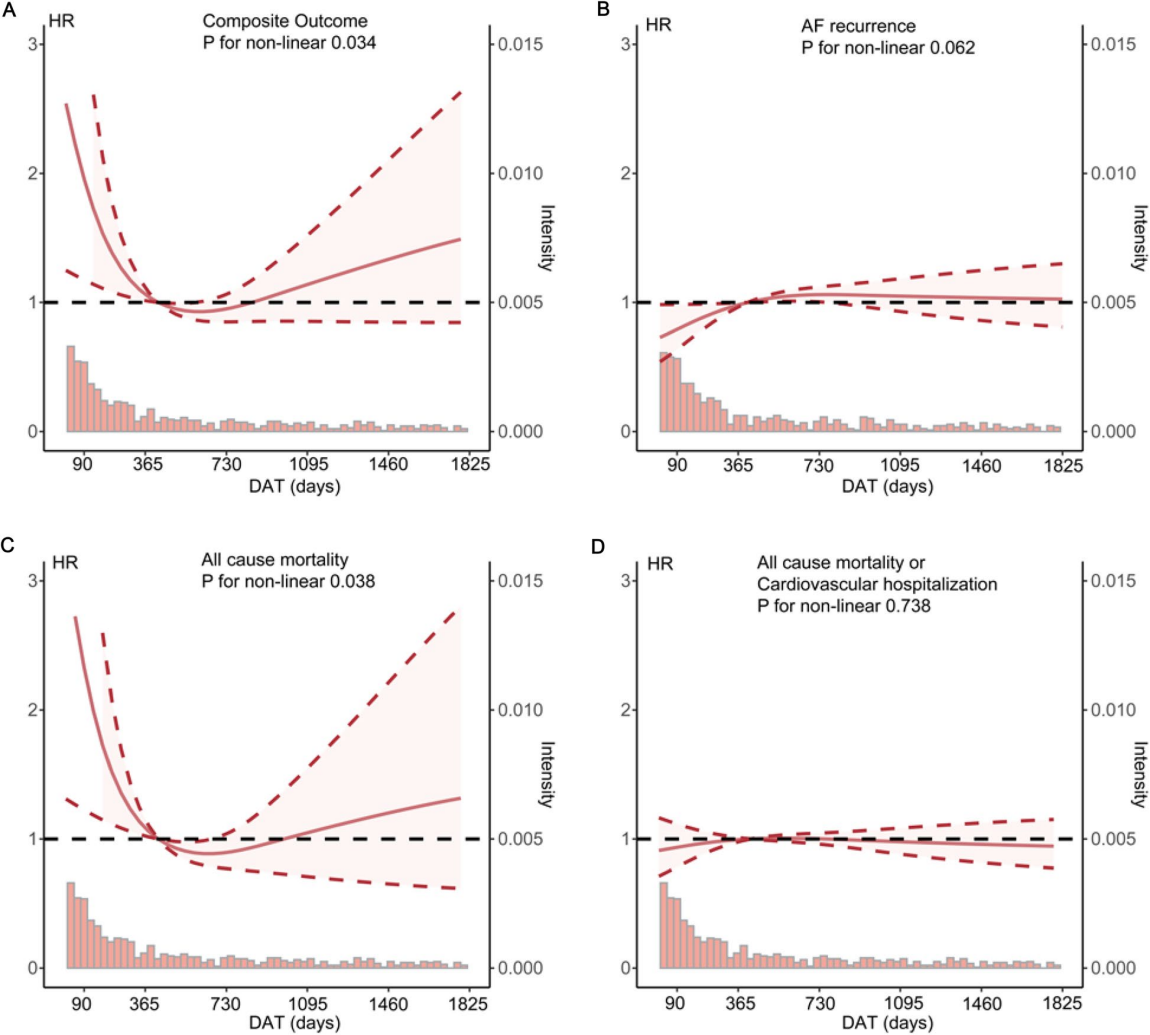
Restricted cubic spline curves of the association between DAT and post-ablation outcomes. **A** Composite outcomes; **B** AF recurrence; **C** All-cause mortality; **D** All-cause mortality or cardiovascular diseases. Adjusted for age(≥ 65 years), sex, CHA_2_DS_2_-VA score, paroxysmal AF and use of oral anticoagulants. The solid line represents the estimated hazard ratios; the shade denotes the 95% confidence interval

An L-shaped association was estimated between DAT and the primary outcome (*P* for non-linear = 0.034), with a rapid decline in hazard ratio (HR) before the inflection point and a gradual increase thereafter. The lowest point was located at a DAT of approximately 1 to 3 years. For AF recurrence, the fitted RCS curve generally showed an upward trend with increasing DAT (*P* for non-linear = 0.062), indicating that earlier ablation (within 1 year of diagnosis) might be associated with a reduced risk of AF recurrence. A similar L-shaped association was depicted for all-cause mortality (*P* for non-linear = 0.038), but not for the outcome of death or CV hospitalisation (*P* for non-linear = 0.738) (Fig. 2).

### Associations between different AFCA timing and clinical outcomes

**Fig. 3:**
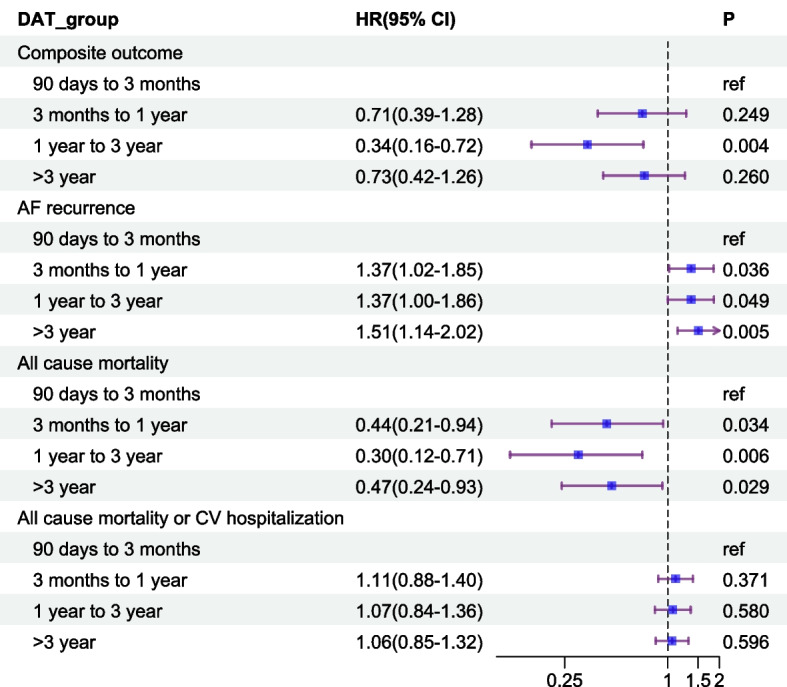
Effects of different ablation timing on post-ablation outcomes based on multivariable Cox regression models. Adjusted for age (≥ 65 years), sex, CHA_2_DS_2_-VA score, paroxysmal AF and use of oral anticoagulants

The DAT was categorised into four groups to explore the associations between AFCA timing and clinical outcomes. Compared to patients in the very early group, the intermediate group demonstrated a significantly lower risk of the primary outcome (HR 0.34, 95% confidence interval (CI): 0.16–0.72), whereas the point estimates were suggestive of lower risk, but these were not statistically significant in the early (HR 0.71, 95% CI: 0.39–1.28) or late (HR 0.73, 95% CI: 0.42–1.26) groups (Fig. 3).

Regarding AF recurrence, the early (HR 1.37, 95% CI: 1.02–1.85), intermediate (HR 1.37, 95% CI: 1.00–1.86) and late (HR 1.51, 95% CI: 1.14–2.02) groups were all associated with significantly increased risks when compared to the very early group. All three groups had significantly lower risks of all-cause mortality compared to the very early group (HR 0.44 for the early group, *P* = 0.034; HR 0.30 for the intermediate group, *P* = 0.006; HR 0.47 for the late group, *P* = 0.029). The association between AFCA timing and the composite outcome of death or CV hospitalisation was not statistically significant (Fig. 3).

A total of 147 patients in our study population were crossed over from the drug therapy arm. Baseline characteristics for patients randomised to ablation and drug arms were shown in Additional file 1: Table S2. Multivariable Cox analyses exclusively for patients in the ablation arm yielded consistent findings (Additional file 1: Fig. S1). After further adjusting for baseline HF history, the results remained aligned with previous Cox models (Additional file 1: Fig. S2). The Kaplan–Meier estimates of cumulative incidence of the primary outcome were included in the supplementary materials (Additional file 1: Fig. S3 and Table S3).

### Subgroup analyses

DAT was further stratified into early ablation (DAT ≤ 1 year) and delayed ablation (DAT > 1 year). The effects of early ablation compared to delayed ablation across different subgroups are shown in Table 2.

**Table 2:** Effect of early ablation (DAT ≤ 1 year) on post-ablation outcomes among different subgroups

Subgroups	HR (95% CI)			
	Composite outcome	AF recurrence	All-cause mortality	All-cause mortality/CV hospitalisation
Age ≥ 65	1.33	0.78	1.57	0.98
(*N* = 751)	(0.84–2.13)	(0.62–0.98)	(0.86–2.86)	(0.81–1.18)
Age < 65	2.64	1.03	2.67	1.04
(*N* = 394)	(0.78–8.92)	(0.69–1.52)	(0.65–10.95)	(0.78–1.38)
***P*-interaction**	**0.242**	**0.316**	**0.454**	**0.782**
HF	1.56	1.20	1.20	1.06
(*N* = 167)	(0.59–4.12)	(0.71–2.01)	(0.42–3.43)	(0.71–1.59)
No HF	1.49	0.78	2.00	0.98
(*N* = 978)	(0.91–2.43)	(0.63–0.96)	(1.03–3.87)	(0.83–1.17)
***P*-interaction**	**0.862**	**0.064**	**0.223**	**0.706**
AAD use	0.86	0.65	0.75	0.89
(*N* = 509)	(0.43–1.73)	(0.48–0.89)	(0.30–1.84)	(0.70–1.14)
No AAD use	2.20	1.00	3.40	1.08
(*N* = 636)	(1.19–4.06)	(0.77–1.29)	(1.45–7.96)	(0.88–1.33)
***P*-interaction**	**0.045**	**0.040**	**0.018**	**0.262**
CCS 0–I	1.08	1.11	1.48	1.01
(*N* = 252)	(0.38–3.06)	(0.68–1.81)	(0.32–6.77)	(0.71–1.44)
CCS II–IV	1.51	0.78	1.72	1.00
(*N* = 839)	(0.93–2.43)	(0.63–0.97)	(0.95–3.11)	(0.84–1.19)
***P*-interaction**	**0.723**	**0.204**	**0.866**	**0.891**
OAC use	1.28	0.78	1.48	0.94
(*N* = 874)	(0.79–2.05)	(0.63–0.97)	(0.82–2.68)	(0.79–1.13)
No OAC use	2.84	1.10	3.16	1.15
(*N* = 266)	(0.89–9.08)	(0.71–1.69)	(0.63–15.74)	(0.84–1.58)
***P*-interaction**	**0.206**	**0.216**	**0.312**	**0.231**

Adjusted for age (≥ 65 years), sex, CHA_2_DS_2_-VA score, paroxysmal AF and use of oral anticoagulants

*HR* hazard ratio, *CI* confidence interval, *AF* atrial fibrillation, *CV* cardiovascular, *DAT* diagnosis to ablation time, *HF* heart failure, *AAD* antiarrhythmic drugs, *CCS* Canadian Cardiovascular Society, *OAC* oral anticoagulants

The effect of early ablation on the primary outcome differed significantly between patients with and without previous or current AAD use. In patients without AAD use, early ablation was associated with an increased risk of the primary outcome, while a reversed trend was observed in those with AAD use (HR 2.20, 95% CI 1.19–4.06 vs. HR 0.86, 95% CI 0.43–1.73, *p*-interaction = 0.045).

Similarly, early ablation was associated with increased risk of all-cause mortality in patients without AAD use but not for those who have received AAD use (HR 3.40, 95% CI 1.45–7.96 vs. HR 0.75, 95% CI 0.30–1.84 respectively, *p*-interaction = 0.018). Patients on AAD showed reduced risks for AF recurrence (HR 0.65, 95% CI 0.48-0.89, *p*-interaction = 0.040), but no significant interactions were found for all-cause mortality or CV hospitalisation (*P*-interaction = 0.262).

The effects of early ablation on both primary and secondary outcomes remained consistent across age (≥ 65 years), HF history, symptom severity and baseline OAC use.

## Discussion

In our study, our main findings are as follows: (i) the impact of DAT on AF recurrence tended to evolve over time, whereby earlier ablation was generally linked to lower recurrence rates; (ii) a L-shaped association appeared to exist between DAT and the composite outcome, indicating that notable CV benefit might be achieved when DAT ranged from 1 to 3 years; and (iii) patients on AAD could potentially benefit from early ablation, whereas prognostic improvement was not significantly observed in those without initial AAD therapy.

Several observational studies and meta-analyses have consistently reported that DAT longer than 1 year is significantly associated with an increased risk of AF recurrence [12, 14, 24, 25]. Another study attempted to identify lower and upper DAT thresholds for reducing AF recurrence, but observed a decreasing trend in AF recurrence risk as DAT shortened without a clear cutoff, while delayed AFCA conferred diminishing benefits, particularly beyond 3 years [26]. In alignment with these findings, we found that AF recurrence risk increases notably when DAT exceeds 1 year.

AF is known to sustain itself through progressive structural, electrical, and contractile remodelling [27]. The electro-anatomical substrate of AF involves dilated atria with small intra-atrial circuits, which may result from changes in connexin expression or atrial structural alterations such as fibrosis [27]. A recent post-hoc analysis of the CABANA trial showed that atrial dilation presented in echocardiography is significantly associated with recurrence [11]. Similarly, atrial fibrosis assessed by delayed-enhancement magnetic resonance imaging has been linked to a higher risk of recurrent arrhythmia [28]. Of note, longer DAT was associated with greater atrial dilation and elevated levels of markers reflecting inflammation and haemodynamic strain, indicating advanced remodelling [29]. Even in early-stage AF patients without comorbidities, atrial remodelling has been observed, implying that short DAT does not preclude arrhythmia progression [30]. The RCS analysis in our study demonstrated a trend of decreasing recurrence with shorter DAT, supporting the possibility that earlier intervention may disrupt the self-perpetuating pathophysiological process of ‘AF begets AF’ and enhance AFCA efficacy.

In addition, prolonged DAT has been associated with a higher likelihood of non-pulmonary vein (non-PV) triggers in patients with persistent AF [31]. These non-PV triggers, particularly when unidentifiable or unablated, are strongly linked to higher recurrence rates following ablation. In our analysis, a large proportion of patients in very early and early AFCA groups had persistent AF, which may partly explain the lower recurrence risk observed with shorter DAT. Furthermore, a high prevalence of persistent AF indicates an increased likelihood of requiring additional ablation procedures. In the CABANA study, the decision to perform additional ablation was at the operator’s discretion [18], leading to variations in ablation strategies and power settings that could influence ablation efficacy and subsequent post-ablation recurrence rates [32]. Given the inherent heterogeneity that could not be adjusted, caution should be warranted when interpreting our results comparing the post-ablation recurrence rates across the four groups.

Contradictory evidence emerged from a RCT comparing early ablation (within 1 month) to delayed ablation (after 12 months of optimised medical therapy), which showed no significant differences in arrhythmia-free survival [33]. While this may be due to limited sample size, it also suggests that atrial remodelling and ablation efficacy are influenced not only by AF progression, but also by other risk factors independently associated with increased recurrence. In previous studies, longer DAT often coincides with older age and a greater burden of comorbidities [34, 35]. In the RCT mentioned, optimal medical therapy may have attenuated the differential effect of early versus delayed ablation on AF recurrence.

Previous studies have typically adopted a 1-year timeframe to assess the timing of catheter ablation in AF patients [3], and some studies have extended this period beyond 3 years [24]. However, evidence is scarce regarding very early ablation within the first year after diagnosis. In our study, we defined ablation within 90 days of AF diagnosis as very early ablation based on previous evidence, which presented that AF diagnosed within 90 days was associated with white matter lesions [21]. Thus, it could be hypothesised that ablation within 90 days of diagnosis might interfere with early subclinical pathophysiological processes, leading to more favourable post-ablation outcomes.

Current evidence on the impact of ablation timing on CV outcomes is inconsistent, partly due to variations in study designs and AF duration. Some observational studies have reported increased risks of major adverse CV events [17], CV hospitalisations [34], all-cause mortality [17, 35], and a modest trend toward higher rates of the composite outcome of HF, stroke or death rates [16] when ablation is delayed beyond 3 years. Also, a recent meta-analysis observed reduced all-cause mortality for patients with DAT ≤ 1 year [12]. From the L-shaped RCS curve in our study, patients with DAT ranging from 1 to 3 years were associated with reduced risks of composite outcomes including death, disabling stroke, serious major bleeding, and cardiac arrest, compared to those who underwent very early AFCA. Our findings suggest that the benefits of earlier ablation in reducing recurrence did not equate to improvements in post-ablation CV outcomes. Nevertheless, given the relatively limited number of events in our study, the results should be interpreted with caution.

Several reasons may explain this discrepancy. First, while AF recurrence patterns have been proved to have prognostic value for post-ablation CV outcomes [36], the patient’s comorbidity status could also influence the post-ablation prognosis. For instance, post-hoc analyses from the CABANA trial have shown that the CV benefits of AFCA may vary by age [37], HF history [38] and cardiac structural abnormalities such as mitral regurgitation [39]. Similarly, the EAST-AFNET 4 trial showed prognostic benefits of an early rhythm control strategy, whereby the study population was characterised by advanced age and multiple comorbidities such as HF, hypertension, diabetes, coronary artery diseases, chronic kidney disease, or left ventricular hypertrophy [3]. In our study, there was a higher prevalence of HF at baseline among patients who received very early ablation compared to other groups. Consequently, the increased risks of post-ablation CV outcomes could be significantly driven by the severity of HF and its management. Second, patients who underwent ablation within 90 days may not have received comprehensive management for AF based on risk stratification, potentially leading to adherence issues to Atrial fibrillation Better Care (ABC) pathway, subsequently increasing the incidence of adverse CV events after ablation [40]. Additionally, there were 84 cases of primary outcome events and 53 cases of death among more than 1100 patients in our analyses. Given the low event rates, our study may not have had sufficient power to assess differences in post-ablation clinical outcomes. Therefore, the results regarding the association between DAT and post-ablation clinical outcomes should be presented only as hypothesis generating.

Among selected patients, current guidelines have suggested AFCA as first-line therapy for paroxysmal or persistent AF to improve symptoms and reduce the AF burden. However, the first-line therapy does not equate to initial treatment. Previous RCTs have shown that ablation might be more effective in maintaining sinus rhythm, compared to drug therapy [6, 41, 42], although frequent cross-over between AAD and AFCA groups could lead to bias. Moreover, the differences in prognostic benefits in clinical outcomes between AFCA and AAD treatment have not been illustrated.

In our analysis, early AFCA appeared to improve CV outcomes in patients with initial AAD treatment, not in those without prior AAD use. It is possibly because the initial AAD use may have halted the remodelling process whilst waiting for ablation. Additionally, the impact of AAD use after AFCA should not be overlooked. Evidence from RCTs has shown that adding AAD therapy to AF ablation improves sinus rhythm maintenance [43, 44], thereby potentially enhancing CV prognosis. Furthermore, real-world data indicate a high prevalence of post-ablation AAD use, underscoring the clinical reliance on both therapeutic strategies for rhythm control. For example, the ARRC-AF study reported that approximately 50% of newly diagnosed AF patients continued AAD treatment after index AFCA [45], and similarly, the EORP cohort reported that about 46% of AF patients remained on AAD following AFCA [46]. However, in the CABANA trial, post-ablation AAD use varied across participating sites depending on the preference of local physicians, making it difficult to precisely determine the timing and types of AAD administered to patients. Consequently, we were unable to adjust for the influence of post-ablation AAD use on CV outcomes in our analyses.

The landscape of ablation techniques is evolving. Previous literature has explored differences in efficacy, safety, and post-ablation prognosis between cryoablation and radiofrequency catheter ablation. Notably, both methods demonstrated comparable 1-year freedom from atrial tachyarrhythmia [47], but cryoablation was associated with a lower incidence of pericardial effusion and tamponade [48]. The Fire and ICE study showed non-inferiority of cryoablation compared to catheter ablation [49]; however, patients who received cryoablation experienced fewer all-cause rehospitalisations and cardiovascular rehospitalisations [50]. Moreover, emerging techniques such as pulsed field ablation have been shown to improve post-ablation recurrence rates while minimising complications such as esophageal and phrenic nerve damage [51]. Our study exclusively focused on patients who underwent catheter ablation, which may limit its generalisability given the continuously evolving advances in clinical practice. Further clinical evidence is needed to establish the relevance of newly developed ablation technologies.

### Limitations

This study has several limitations. First, as a post-hoc analysis of a RCT, selection bias was unavoidable. It is important to highlight that in our analyses, patients who received very early AFCA exhibited a higher prevalence of HF at baseline compared to other groups, which could not represent the patients’ characteristics in real-world scenarios. Second, the duration from AF onset to ablation is difficult to define precisely in clinical practice, especially for PAF and asymptomatic AF. Patients may have experienced AF prior to noticing related symptoms or having it detected during routine examinations. In our study, AF onset was established as the date of the initial documented episode, leading to an underestimation of the DAT and subsequently confounding the comparison of post-ablation outcomes for patients with different DAT. Additionally, in the CABANA study, ECG recordings were based on symptom-triggered and regular monitoring, which could not adequately account for AF burden. However, the EARLY-AF study employed more efficient prolonged monitoring with an implanted loop recorder and demonstrated that the burden of AF recurrence is almost three times higher than that associated with symptomatic AF episodes. Thus, the recurrence rates observed in our study may have underestimated the true AF burden, leading to a diminished evaluation of the effect of DAT on post-ablation recurrence rates.

## Conclusions

In this post-hoc analysis, we presented potential temporal trends of the associations between DAT and post-ablation outcomes. Earlier ablation was generally linked to lower recurrence rates, but the benefits of AFCA in reducing AF recurrence might not transform into prognostic benefits. Acceptable delayed AFCA could improve CV outcomes and reduce mortality, despite increased AF recurrence. The significant effects associated with improved CV outcomes might be achieved when DAT roughly ranged from 1 to 3 years, and with initial AAD use. Our findings indicate that timely intervention in AF management is crucial for slowing AF progression; meanwhile, the optimal treatment combining AADs and AFCA may provide additional CV benefits. Given the post-hoc nature of the analyses and the limited number of outcome events, this study provides hypothesis-generating evidence that warrants further investigation in prospective cohorts.

## Supplementary Information

Additional file 1: Table S1. Proportions of patients with previous or current use of specific antiarrhythmic drugs at baseline. Table S2. Baseline characteristics for patients randomised to ablation and drug arms. Table S3. Kaplan–Meier estimates of cumulative incidence of the primary outcome at different timepoints during the follow-up. Figure S1. Effects of different ablation timing on post-ablation outcomes based on multivariable Cox regression models among patients randomised to ablation arm. Figure S2. Effects of different ablation timing on post-ablation outcomes based on multivariable Cox regression models. Figure S3. Kaplan–Meier estimates of the cumulative incidence of the primary outcome.

## Data Availability

The data supporting the findings of this study is available in the National Heart Lung, and Blood Institute BioLiNCC data repository, https://biolincc.nhlbi.nih.gov/home.

## Abbreviations

AF: Atrial fibrillation
CV: Cardiovascular
AFCA: Atrial fibrillation catheter ablation
AAD: Antiarrhythmic drug
DAT: Diagnosis to ablation time
HF: Heart failure
CABANA: Catheter Ablation vs. Antiarrhythmic Drug Therapy for Atrial fibrillation
RCT: Randomised controlled trial
ECG: Electrocardiogram
IQR: Interquartile range
RCS: Restricted cubic spline
PAF: Paroxysmal atrial fibrillation
OAC: Oral anticoagulants
HR: Hazard ratio
CI: Confidence interval
PV: Pulmonary vein
EAST-AFNET4: Early Treatment of Atrial Fibrillation for Stroke Prevention Trial
