# Supplementary material for: Impact of diagnosis to ablation time on clinical outcomes in patients with atrial fibrillation: post hoc analysis of the CABANA trial
Source: BMC Med. 2026 Jan 12;24:81. doi: 10.1186/s12916-026-04615-3 (PMC12888738; doi:10.1186/s12916-026-04615-3)
Supplement: Supplementary file 1 — Additional file 1: Table S1. Proportions of patients with previous or current use of specific antiarrhythmic drugs at baseline. Table S2. Baseline characteristics for patients randomised to ablation and drug arms. Table S3. Kaplan–Meier estimates of cumulative incidence of the primary outcome at different timepoints during the follow-up. Figure S1. Effects of different ablation timing on post-ablation outcomes based on multivariable Cox regression models among patients randomised to ablation arm. Figure S2. Effects of different ablation timing on post-ablation outcomes based on multivariable Cox regression models. Figure S3. Kaplan–Meier estimates of the cumulative incidence of the primary outcome. [file 12916_2026_4615_MOESM1_ESM.docx]

**Impact of diagnosis to ablation time on clinical outcomes in patients with atrial fibrillation: post-hoc analysis of the CABANA trial**

Manlin Zhao, MD^1,2^#, Yang Chen, MD^2,3^ #, Mingxiao Li, MD^1^, Chao Jiang, MD^1^, Zhen Wang, MD^1^, Hongyu Liu, MD^2,4^, Liu He, PhD^1^, Caihua Sang, MD^1^, Xin Du, MD^1,5,6^, Jianzeng Dong, MD^1,5,7^, Douglas L Packer, MD^,8^, Changsheng Ma, MD^1,5,9^*, Gregory Y. H. Lip, MD^2,10,11^ *

Supplementary material

**Table S1. Proportions of patients with previous or current use of specific antiarrhythmic drugs at baseline.**

Values are presented as n (%).

Abbreviations: AFCA=Atrial fibrillation catheter ablation.

|  | Very Early AFCA (0≤DAT≤90 days) (N=242) | Early AFCA (90 days＜DAT≤1 year) (N=296) | Intermediate AFCA (1 year＜DAT≤3 years) (N=248) | Late AFCA (DAT>3 years)  (N=359) | P  value |
| --- | --- | --- | --- | --- | --- |
| Amiodarone (%) | 20 (8.3) | 29 (9.8) | 24 (9.7) | 53 (14.8) | 0.048 |
| Propafenone (%) | 15 (6.2) | 12 (4.1) | 20 (8.1) | 50 (13.9) | 0.502 |
| Doronedarone (%) | 11 (4.5) | 19 (6.4) | 20 (8.1) | 33 (9.2) | <0.001 |
| Sotalol (%) | 15 (6.2) | 39 (13.2) | 25 (10.1) | 62 (17.3) | 0.001 |
| Dofetilide (%) | 3 (1.2) | 9 (3.0) | 5 (2.0) | 7 (1.9) | 0.533 |

**Table S2. Baseline characteristics for patients randomized to ablation and drug arms.**

Abbreviations: DAT=diagnosis to ablation time, BMI= body mass index, AF= atrial fibrillation, CCS= Canadian cardiovascular society, CVA= cerebral vascular accident, TIA=transit ischemic attack, LVEF=left ventricular ejection fraction, AAD=antiarrhythmic drug.

|  | Randomized to Ablation Arm (N=998) | Randomized to Drug Arm (N=147) | P  value |
| --- | --- | --- | --- |
| Age, years | 67.0 [61.0, 72.0] | 68.0 [61.0, 71.0] | 0.420 |
| Male gender (%) | 629 (63.0) | 96 (65.3) | 0.657 |
| BMI, kg/m^2^ | 30.4[26.4, 33.7] | 30.0 [26.6, 34.3] | 0.793 |
| AF severity (CCS class) |  |  | 0.276 |
| Class 0 | 90 (9.1) | 13 (8.9) |  |
| Class I | 147 (14.8) | 16 (11.0) |  |
| Class II | 316 (31.8) | 39 (26.7) |  |
| Class III | 369 (37.1) | 63 (43.2) |  |
| Class IV | 72 (7.2) | 15 (10.3) |  |
| Hypertension | 786 (78.8) | 116 (78.9) | 1.000 |
| Diabetes | 249 (24.9) | 42 (28.6) | 0.401 |
| Prior CVA or TIA | 107 (10.7) | 18 (12.2) | 0.681 |
| Coronary artery disease | 189 (18.9) | 35 (23.8) | 0.201 |
| Perivascular diseases | 39 (3.9) | 10 (6.8) | 0.161 |
| Sleep apnea | 242 (24.2) | 34 (23.1) | 0.847 |
| Family history of AF | 114 (11.4) | 19 (13.0) | 0.679 |
| Heart failure | 152 (15.2) | 15 (10.2) | 0.137 |
| LVEF≤35% | 32 (4.8) | 5 (5.7) | 0.908 |
| AF type at enrolment |  |  | 0.054 |
| Paroxysmal | 428 (42.9) | 74 (50.3) |  |
| Persistent | 467 (46.8) | 66 (44.9) |  |
| Long-standing persistent | 103 (10.3) | 7 (4.8) |  |
| CHA_2_DS_2_-VASc score | 3.0 [2.0, 3.0] | 3.0 [2.0, 4.0] | 0.603 |
| CHA_2_DS_2_-VASc score≥2 | 811 (81.3) | 125 (85.0) | 0.322 |
| CHA_2_DS_2_-VA score | 2.0 [2.0, 3.0] | 2.0 [2.0, 3.0] | 0.530 |
| CHA_2_DS_2_-VA score≥2 | 756 (75.8) | 119 (81.0) | 0.200 |
| Current/previous OAC use | 757 (75.9) | 106 (72.1) | 0.378 |
| Current/previous AAD use | 438 (43.9) | 71 (48.3) | 0.360 |

**Figure S1. Effects of different ablation timing on post-ablation outcomes based on multivariable Cox regression models among patients randomized to ablation arm.**

Adjusted for age (≥65 years), sex, CHA_2_DS_2_-VA score, paroxysmal AF and use of oral anticoagulants.

**
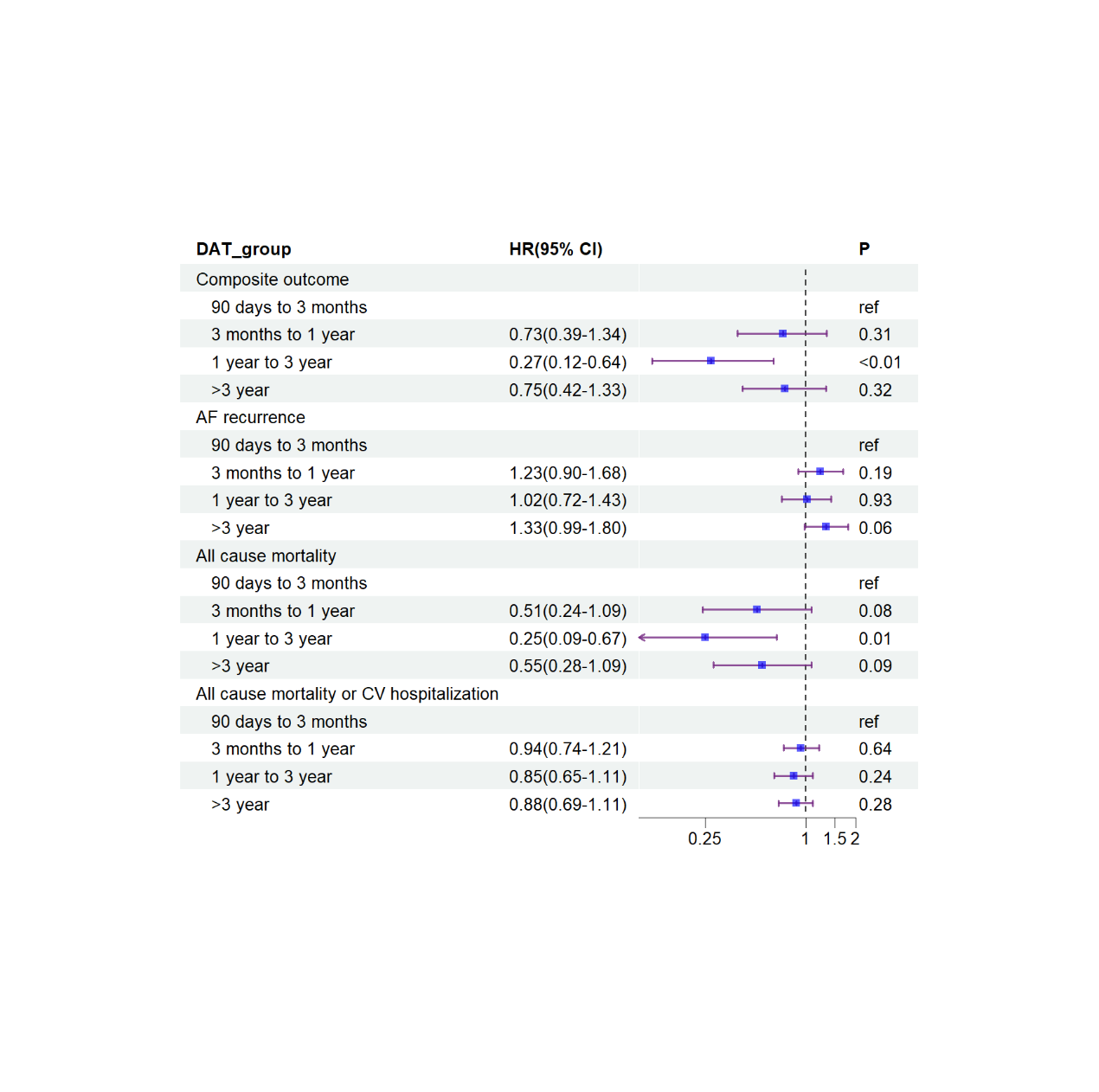
**

**Figure S2. Effects of different ablation timing on post-ablation outcomes based on multivariable Cox regression models.**

Adjusted for age (≥65 years), sex, CHA_2_DS_2_-VA score, paroxysmal AF and use of oral anticoagulants, heart failure.

**
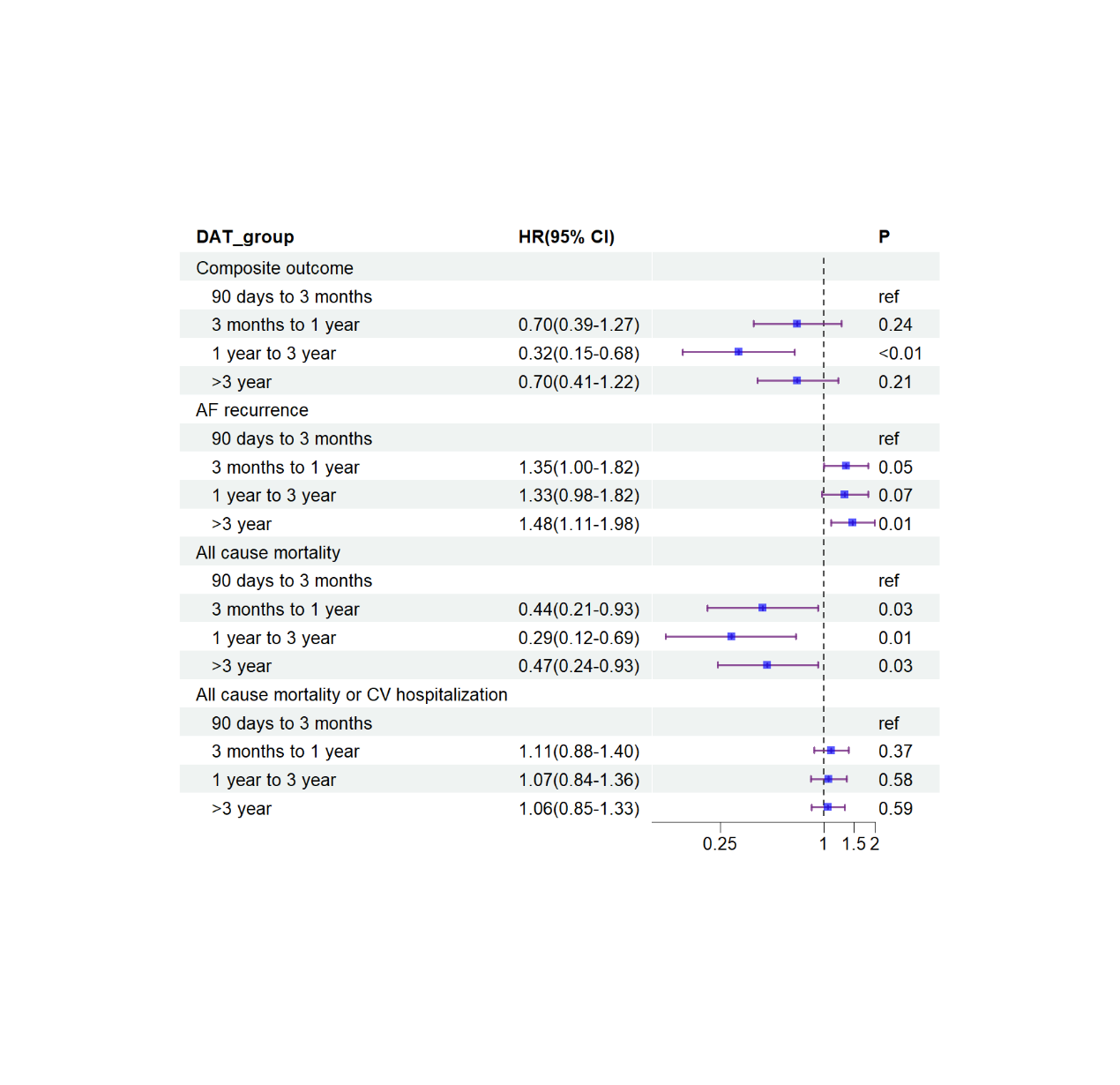
**

**Figure S3. Kaplan-Meier estimates of the cumulative incidence of the primary outcome.**

The study participants were categorized into groups as follows: Group A: very early AFCA (0≤DAT≤90 days); Group B: early AFCA (90 days＜DAT≤1 year); Group C: Intermediate AFCA (1 year＜DAT≤3 years); Group D: Late AFCA (DAT>3 years).

Abbreviations: DAT=diagnosis to ablation time, AFCA= atrial fibrillation catheter ablation.

*
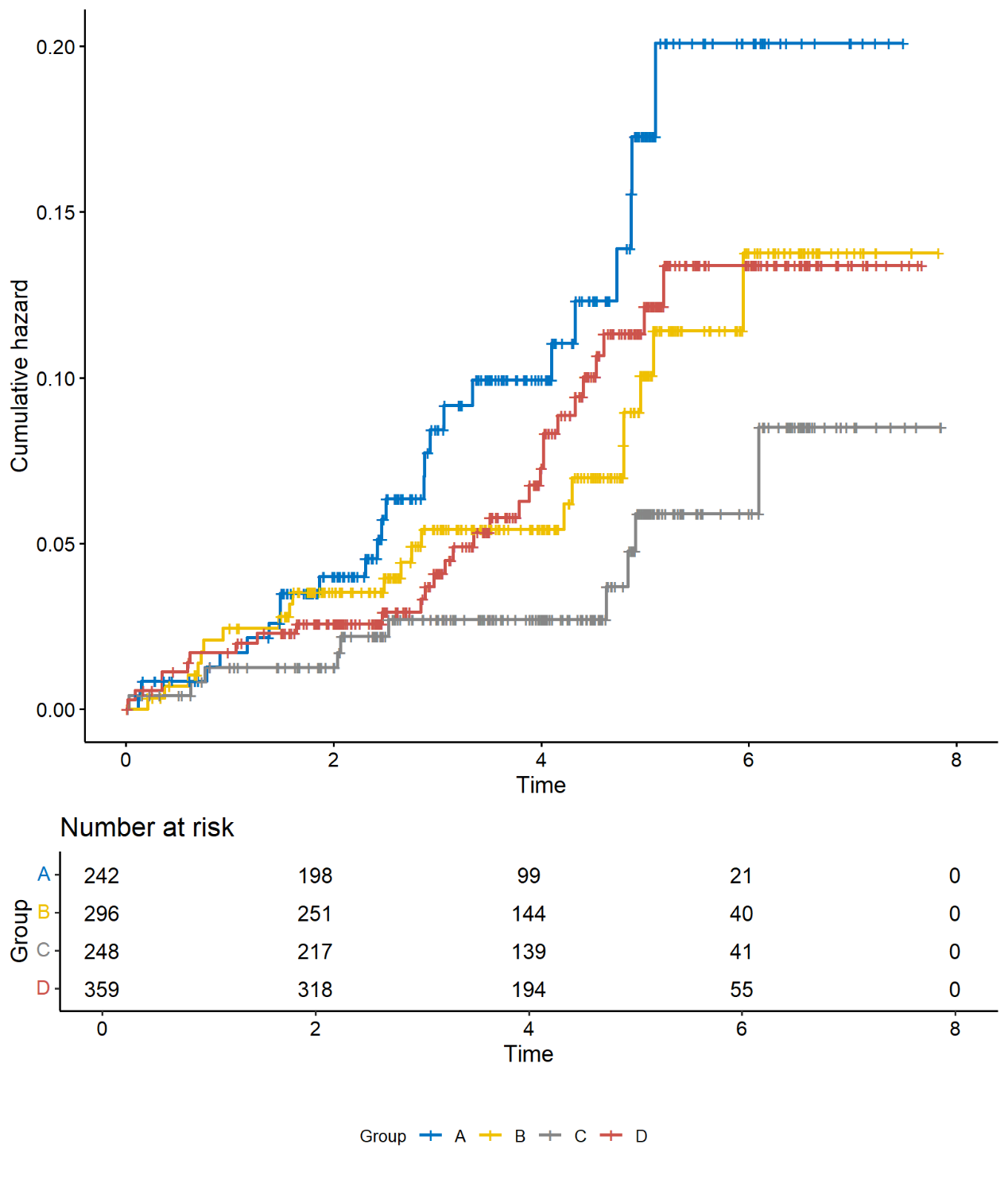
*

**Table S3. Kaplan-Meier estimates of cumulative incidence of the primary outcome at different timepoints during the follow-up.**

Kaplan-Meier Event rates at different timepoints presented with 95% confidence interval.

Abbreviations: DAT=diagnosis to ablation time, AFCA= atrial fibrillation catheter ablation.

| Kaplan-Meier Event rates at different timepoints (%) | Very Early AFCA (0≤DAT≤90 days) | Early AFCA (90 days＜DAT≤1 year) | Intermediate AFCA (1 year＜DAT≤3 years) | Late AFCA (DAT>3 years) |
| --- | --- | --- | --- | --- |
| Year one | 1.69  (0.03-3.32) | 2.41  (0.63-4.16) | 1.24  (0.00-2.62) | 1.68  (0.34-3.01) |
| Year two | 3.91  (1.37-639) | 3.47  (1.33-5.56) | 1.24  (0.00-2.62) | 2.54  (0.89-4.17) |
| Year three | 8.09  (4.13-11.88) | 5.29  (2.52-7.97) | 2.67  (0.53-4.76) | 4.00  (1.83-6.13) |
| Year four | 9.46  (5.1-13.62) | 5.29  (2.52-7.97) | 2.67  (0.53-4.76) | 7.02  (3.93-10.00) |
| Year five | 15.87  (8.83-22.37) | 9.57  (4.93-13.98) | 5.73  (1.66-9.63) | 11.45  (7.16-15.54) |
